# Investigation on the Anaphylaxis and Anti-Digestive Stable Peptides Identification of Ultrasound-Treated α-Lactalbumin during In-Vitro Gastroduodenal Digestion

**DOI:** 10.3390/foods10112760

**Published:** 2021-11-10

**Authors:** Xumei Wang, Zongcai Tu, Guangxian Liu, Hui Wang, Yueming Hu, Tao Huang

**Affiliations:** 1 State Key Laboratory of Food Science and Technology, Nanchang University, 235 East Nanjing Road, Nanchang 330047, China; 13184579152@163.com (X.W.); tuzc_mail@aliyun.com (Z.T.); huyueming@ncu.edu.cn (Y.H.); 2 National R&D Center for Freshwater Fish Processing, Jiangxi Normal University, Nanchang 330022, China; 3 Engineering Research Center of Freshwater Fish High-Value Utilization of Jiangxi Province, Jiangxi Normal University, Nanchang 330022, China; 4 Institute of Food Science and Technology, Jiangxi Academy of Agricultural Sciences, Nanchang 330200, China; Liugx178@163.com; 5 College of Food and Pharmaceutical Sciences, Ningbo University, Ningbo 315800, China; huangtao@nbu.edu.cn

**Keywords:** α-lactalbumin, ultrasound, in-vitro gastroduodenal digestion, anaphylaxis, anti-digestive stable peptides, high-resolution mass spectrometry

## Abstract

Our previous studies indicated that ultrasound treatment can increase the anaphylaxis of protein. However, investigation on the anaphylaxis changes of ultrasound-treated α-lactalbumin (ALA) during digestion is lacking. The anaphylaxis of ultrasound-treated ALA and its digesta was investigated. The anti-digestive stable peptides were identified by high-resolution mass spectrometry. Ultrasound induced the tertiary structure of ALA to unfold and increased its anaphylaxis. During digestion, the anaphylaxis of both gastric and gastroduodenal digesta was further increased. There are two reasons for this phenomenon. On the one hand, linear epitopes played an important role in affecting anaphylaxis compared with the conformational epitope, and some linear epitopes were still retained on the anti-digestive stable peptides produced after gastroduodenal digestion, resulting in increased anaphylaxis after digestion. On the other hand, the presence of intact ALA molecules after digestion still remained strong anaphylaxis. Compared with the digesta of untreated ALA, the digesta of ultrasound-treated ALA possessed higher anaphylaxis. The results indicated that ultrasound increased the anaphylaxis of ALA during digestion.

## 1. Introduction

Novel non-thermal food processing technologies are emerging and prospectively maintaining the sensory properties of foodstuff that traditional processing methods such as heating and boiling cannot match [1]. The high-intensity ultrasound technique is specified as a sound wave frequency of 20–100 kHz which is widely applied to microorganism inactivation, components extraction, and cell walls crushing due to its nutrient retention and appreciable acceptability [2]. Recently, ultrasound has also attracted desirable attention for its ability to improve the functional properties (emulsification, foaming properties, and oxidation resistance) of proteins [3,4]. Dong et al. [5]. indicated that the antioxidant activity of ultrasound-processed tropomyosin increases with the prolongation of ultrasound action time. Ultrasound has also drawn the close attention of researchers for its application in protein anaphylactogens [6]. 

Milk and milk-containing food products are recognized as a necessity in western countries. However, the prevalence of worldwide anaphylactic diseases such as food allergy has increased [7]. Among food-induced anaphylaxis, cows’ milk allergy (CMA) is an anaphylactic response arising from whey protein such as α-lactalbumin (ALA), primarily mediated by IgE, which can arouse cell degranulation, and then trigger diarrhea, gastroduodenal bleeding, asthma, etc. [8,9]. When breastfeeding cannot be achieved, infant formulas based on cows’ milk are an indispensable alternative for infant feeding [10]. Population-based studies have manifested that the prevalence of CMA in infants ranges from 2–3%, and that in adults is below 0.5%, indicating infants are more likely to suffer from severe anaphylactic diseases [11,12]. Small milk protein ALA in cows’ milk is the main anaphylactogen and a globular protein consisting of 123 amino acids with a molecular weight of 14.4 kDa [13]. Many methods have focused on improving the physicochemical and functional properties of ALA [14,15,16,17,18]. However, limited information is available on the influence of ultrasound treatment on the anaphylaxis potential of ALA.

Comprehending the digestion of protein anaphylactogens has become of particular importance due to digestion playing a significant role in ascertaining the potential anaphylaxis of protein. Meanwhile, the influence of processing technologies on the gastroduodenal digestion of anaphylactic proteins is another factor that deserves to be considered. Given the immature digestive system of infants, infants present a higher incidence rate of CMA than adults [19,20]. Therefore, the immature gastroduodenal tract of infants should be taken into account for systematically understanding the digestion of protein anaphylactogens. There is evidence that traditional heat processing contributes to unfolding the structure of ovotransferrin, thus exposing antibody-binding epitopes and increasing anaphylaxis [21]. A contradictory conclusion has been reported that irradiation-induced damage to the structure of ALA is the reason for the reduced anaphylaxis of ALA [17,22]. One potential explanation might be that the intensity of food processing technologies and the stability of proteins result in the susceptibility of antibody-binding epitopes to unfold or damage. Our previous study reported the IgG/IgE-binding properties of ultrasound-treated ovalbumin and discovered that ultrasound could raise anaphylaxis through unfolding its tertiary structure [23]. However, the anaphylaxis changes of ultrasound-treated ALA during in-vitro gastroduodenal digestion are unknown. 

Overall, the objective of this study was to research the anaphylaxis changes of ultrasound-treated ALA during in-vitro gastroduodenal digestion in infants and to identify the anti-digestive stable peptides. Considering the unfolding effect of ultrasound on ALA, ALA was treated at the intensity of 50 and 100 W/cm^2^. The structural characterization was examined through fluorescence spectroscopy and tricine SDS PAGE. The anaphylaxis was evaluated through indirect competitive enzyme-linked immunosorbent assay (icELISA) and degranulation assay. The digesta was isolated by gel filtration chromatography. Finally, the anti-digestive stable peptides were identified by a high-performance liquid chromatography system coupled to tandem mass spectrometry (LC-MS/MS). 

## 2. Materials and Methods 

### 2.1. Materials

Bovine ALA (L6010, ≥85%), sodium glycodeoxycholate (G9910), and sodium taurocholate (S0900000) were obtained from Sigma-Aldrich (St.Louis, MO, USA). 4-(2-Aminoethyl) benzenesulfonyl fluoride hydrochloride (D131214) was purchased from Aladdin (Shanghai, China). Pepsin (P8160, 1:10,000), trypsin (T8150, 1:250), α-chymotrypsin (YZ-1134007, 1:1200), and 3, 3′, 5, 5′-tetramethylbenzidine (TMB) two-component substrate solution (PR1210) were supplied by Solarbio (Beijing, China). Horseradish peroxidase (HRP)-labeled goat anti-rabbit IgG (BL003A) was from Biosharp (Shenzhen, China). CMA patients’ sera (pooled sera including 10 patients) was afforded from Plasma Lab International (Everett, WA, USA). The detailed information of the 10 patients can be found in Supplementary Table S1. HRP-labeled goat anti-human IgE (A9667) was bought from Biocheck (San Francisco, CA, USA). Human ELISA kits for β-hexosaminidase (β-Hex), interleukin 6 (IL-6), and histamine were supplied by Meimian (Yancheng, China). The KU812 cells were obtained from iCell Bioscience (Shanghai, China).

### 2.2. Preparing of Rabbit Anti-ALA IgG

Male Japanese rabbits (three months, permission number was SCXK (Gan) 2014-0005) were purchased from Longping (Nanchang, Jiangxi), which were used to prepare polyclonal rabbit anti-ALA IgG. All procedures were authorized by the ethics committee of Nanchang University (approval number 0064257) and were carried out according to Chinese guidelines for animal welfare (GB/T35892-2018). After acclimatizing in an environmentally controlled breeding room for 7 days, the rabbits were intravenously injected with 0.8 mg/mL of ALA (1 mL) emulsified with the same volume of Freund’s complete adjuvant for the first time. After that, ALA was emulsified by Freund’s incomplete adjuvant at 1:1 (*v*/*v*), and the subsequent booster immunization was carried out at a 6-day interval with a dose of 0.5 mg per rabbit. After the rabbits were anesthetized, the plasma was collected and the rabbit’s anti-ALA IgG serum was separated by centrifuging below 4 °C and stored at −80 °C.

### 2.3. Ultrasound Treatment

Considering that ALA accounts for 5% of milk proteins, bovine ALA was suspended in phosphate buffer (pH 7.4, 0.05 mol/L) to a concentration of 1 mg/mL, and 20 mL of ALA solution was treated at a gradually increased intensity (50 and 100 W/cm^2^) in an ultrasound processor (Scientz-IID, Ningbo, China) equipped with a 3 mm microtip probe. The ultrasound treatment was performed for 10 min with the pulsation of 2 s on, and 2 s off. The samples were in ice-bath conditions to ensure the temperature was below 20 °C. Untreated ALA was named ALA-0. Ultrasound-treated ALA at 50 and 100 W/cm^2^ were named ALA-50 and ALA-100, respectively. ALA-0, ALA-50, and ALA-100 were collectively referred to as substrate samples. Other milk components (e.g., fats) that could influence the ultrasound treatment of ALA were not investigated in this study.

### 2.4. Gastroduodenal Digestion In-Vitro

To better simulate the physiological parameters of the infant gastroduodenal tract, the digestive behavior of substrate samples was performed by a previous method, with some adaptations [24]. In the process of simulated gastric juice (SGJ) digestion, substrate samples were mixed with 10 mL of SGJ (Ph = 3.0) embodied with pepsin (22.75 U/mg sample) and NaCl (0.15 mol/L). After performing for 2 h at 37 °C, the gastric digestive behavior was suspended by monitoring the pH to 7.0. Afterward, 10 mL of simulated duodenal juice (SDJ) was added to digest for 2 h, and the components of SDJ were as follows: 2 mmol/L of bile salts (sodium glycodeoxycholate and sodium taurocholate), 3.45 U trypsin/mg sample, and 0.04 U chymotrypsin/mg sample. It was stopped by adding 4 mmol/L of protease inhibitor 4-(2-Aminoethyl) benzenesulfonyl fluoride hydrochloride. Samples were placed below −20 °C until used. ALA-0, ALA-50, and ALA-100 digested by SGJ were named ALA-0G, ALA-50G, and ALA-100G, respectively, and ALA-0G, ALA-50G, and ALA-100G were called gastric digesta; ALA-0, ALA-50, and ALA-100 digested by SGJ followed SDJ was denoted as ALA-0D, ALA-50D, and ALA-100D, respectively, and ALA-0D, ALA-50D, and ALA-100D were called gastroduodenal digesta. 

### 2.5. Structure Analysis after Ultrasound Treatment

#### 2.5.1. Fluorescence Spectrophotometer

The intrinsic fluorescence was analyzed according to a previous report with some modifications [25]. 0.5 mg/mL of substrate samples, gastric digesta, and gastroduodenal digesta were prepared for intrinsic fluorescence spectra detection by a fluorescence spectrophotometer (Hitachi F-7000, Tokyo, Japan). The excitation wavelength was 280 nm, and the emission spectrum was recorded from 300 nm to 600 nm, with 2 nm bandpass filters in both excitation and emission.

#### 2.5.2. Analysis of Tricine SDS PAGE

Tricine SDS PAGE analysis was carried out according to a previous study with some modifications [26]. To detect the pattern of ALA-0, ALA-50, ALA-100, ALA-0G, ALA-50G, ALA-100G, ALA-0D, ALA-50D, and ALA-100D, separating gel (16.5%), spacer gel (10%), and stacking gel (4%) were prepared for electrophoresis (15 µL of samples were applied) with an initial voltage of 30 V (1–2 h) until the indicator entered separating gel, then 100 V was employed. Following electrophoresis, gels were fixed with fixation fluid (0.5% glutaraldehyde in 30% alcohol) for 30 min and dyed with coomassie brilliant blue R-250 for 20 min, and finally destained until the background was transparent. The samples’ distribution in tricine SDS PAGE gel was transferred to a nitrocellulose membrane for western blotting. The analysis of western blotting was carried out according to a method previously described with some modifications [27].

### 2.6. Indirect Competitive ELISA

The IgG/IgE-binding abilities were analyzed by icELISA with rabbit anti-ALA IgG and CMA patients’ sera. Briefly, the incubation circumstance was at 37 °C for 1 h and the washing solution was PBST (containing 0.05% Tween-20). The incubation sequence was as follows: each well of the microplate was coated with 2 µg/mL of native ALA (100 µL/well); 1% (*w*/*v*) of fish gelatin blocking solution (250 µL) was blocked; 50 µL of rabbit anti-ALA IgG (1:10,000) or CMA patients’ sera (1:20) and 50 µL of samples were blended in the wells; HRP-labeled goat anti-rabbit IgG (1:5000) or HRP-labeled goat anti-human IgE (1:800) was followed; TMB two-component substrate solution was used for color reaction (15 min); sulfuric acid (2 mol/L) was added to stop the reaction. The binding rate was calculated following the equation previously described [28]. The inhibition rate was calculated as:

$$The\;inhibition\;rate\;\left(\%\right)=\frac{B_{0}-B}{B_{0}-B_{c}}\times 100\%$$

where *B* and *B*_0_ are the OD values with and without samples, respectively. *B_c_* was the OD values without rabbit anti-ALA IgG or CMA patients’ sera. IC50 is the concentration of samples that causes a 50% inhibition of IgG recognition (μg/mL). Each sample was performed in triplicate.

### 2.7. Degranulation Assay in KU812 Cells

The KU812 cells were maintained in the medium RPMI-1640, which contained 10% of fetal bovine sera and 105 U/L of penicillin/streptomycin. The culture condition was 37 °C with CO_2_ (5%). The sensitizing condition was as follows: KU812 cells were activated by 10 μL of patients’ sera (24 h); then 100 μL/well of samples (0.5 mg/mL) were added for activation (3 h). The degranulation assay (the release of β-Hex, IL-6, and histamine) was analyzed by ELISA assays, following the instruction of commercial kits. 

### 2.8. Gel Filtration Chromatography Isolation

Gastroduodenal digesta were isolated according to the previous study with some modifications [29]. Freeze-dried ALA-0D and ALA-100D were dissolved in ultrapure water (2 mL). After being filtered through nylon filter membrane (0.45 mm), ALA-0D and ALA-100D were loaded onto Sephadex G-25 column (Φ 16 mm × 50 cm). An HD-21–88 ultraviolet detector (QiTe, Shanghai, China) coupled with an automatic fraction collector (QiTe, Shanghai, China) was used. The isolated condition was as follows: the eluent was ultrapure water; the flow rate was 0.4 mL/min; the elution solution was collected every 8 min; the detected wavelength was 220 nm; the detection sensitivity was 0.5 A.

### 2.9. Identification of Anti-Digestive Stable Peptides by HPLC-MS/MS

The anti-digestive stable peptides were identified by HPLC-Orbitrap Q-Exactive HF mass spectrometer (Thermo Fisher Scientific, Waltham, MA, USA). The HPLC condition was as following: the analytical column was AcclaimR PepMap RSLC (50 μm × 15 cm, C18, 2 μm, 100 Å); the flow rate was 220 nl/min; the mobile phase was 0.1% formic acid of aqueous solution (A) and 0.1% formic acid of acetonitrile solution (B); the gradient condition was set to 0 min (96% A, 4% B), 2 min (88% A, 12% B), 25 min (78% A, 22% B), 32 min (68% A, 32% B), 37 min (25% A, 75% B), 40 min (25% A, 75% B). The MS/MS condition was as follows: automatic switching between MS and MS/MS scans used the top-20 method; the scanning range was limited to 350–1650 *m*/*z*; peptide fragmentation was performed via higher-energy collision dissociation (HCD). The parent ion map was analyzed by Xcalibur software. Bos taurus Reference Database (Proteome ID: UP000009136) was used.

## 3. Results and Discussion

### 3.1. Structural Characterization 

#### 3.1.1. Structure of Ultrasound-Treated ALA 

**Figure 1:**
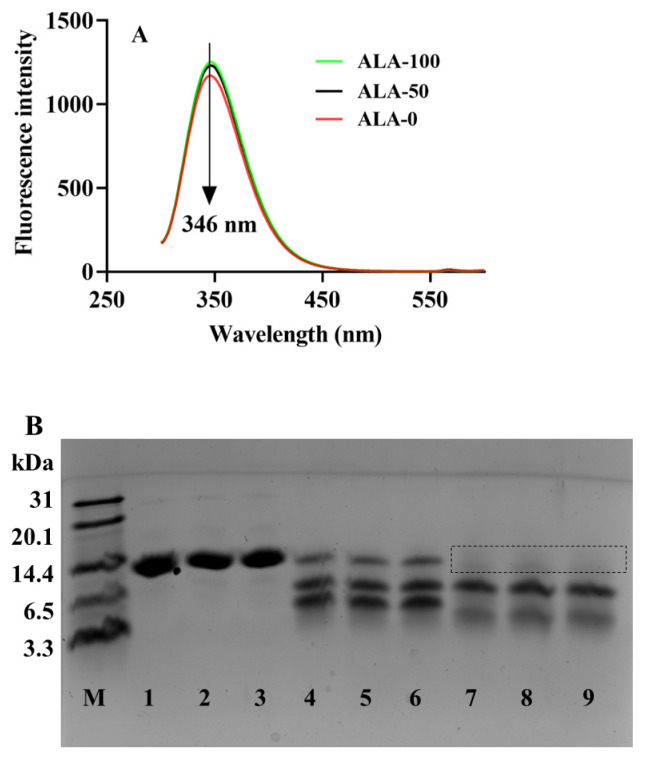
Influence of ultrasound on intrinsic fluorescence spectra of samples (**A**); the tricine SDS PAGE pattern (**B**) of ALA-0 (Lane 1), ALA-50 (Lane 2), ALA-100 (Lane 3), ALA-0G (Lane 4), ALA-50G (Lane 5), ALA-100G (Lane 6), ALA-0D (Lane 7), ALA-50D (Lane 8), and ALA-100D (Lane 9). ALA-0: untreated ALA, ALA-50: ultrasound-treated ALA at 50 W/cm^2^, ALA-100: ultrasound-treated ALA at 100 W/cm^2^. ALA-0G, ALA-50G, and ALA-100G: ALA-0, ALA-50, and ALA-100 digested by simulated gastric juice. ALA-0D, ALA-50D, and ALA-100D: ALA-0, ALA-50, and ALA-100 digested by simulated gastric juice followed simulated duodenal juice.

Aromatic amino acids in ALA, especially tryptophan, are the basis of intrinsic fluorescence. The intrinsic fluorescence spectra evolution during ultrasound treatment of ALA was analyzed, due to the appropriability of intrinsic fluorescence spectra for investigating the tertiary structure changes in proteins. The fluorescence emission intensity was detected at a wavelength range of 200–600 nm. When excited at 280 nm, ALA-0 presented a fluorescence emission maximum (λmax) at 346 nm (Figure 1A). ALA-50 and ALA-100 displayed an increase in fluorescence intensity at the same λmax with an increase in ultrasound intensity. The results indicated that ultrasound could induce the tertiary structure of ALA unfolding and expose aromatic amino acids, thus enhancing the fluorescence intensity. A similar phenomenon was observed by Shao et al. [30].

The molecular weight distribution of samples obtained from the tricine SDS PAGE pattern is outlined in Figure 1B. Obvious bands of ALA-0, ALA-50, and ALA-100 approaching 14.4 kDa were visible (Lanes 1–3 in Figure 1B), and the three bands (Lanes 1–3) had a similar migration distance. No major changes of lanes 1–3 were discovered in the tricine SDS PAGE pattern, revealing ultrasound treatment could not cause ALA aggregate to polymers or degrade to low-molecular-weight products [31]. Therefore, combined with the analysis of intrinsic fluorescence spectra, after ultrasound treatment, the tertiary structure of ALA was unfolded but the primary structure was not destroyed. 

#### 3.1.2. Structure Characteristic of Ultrasound-Treated ALA during Gastroduodenal Digestion In-Vitro

To investigate the variation in the structure of ALA after gastric/gastroduodenal digestion, ALA-0G, ALA-50G, ALA-100G, ALA-0D, ALA-50D, and ALA-100D was analyzed by tricine SDS PAGE (Lanes 4–9 in Figure 1B). In comparison to substrate samples, pepsin hydrolysis caused the tricine SDS-PAGE pattern of gastric digesta (Lanes 4–6 in Figure 1B) to form three hydrolysis fragments (distributed in 3.3–14.4 kDa), implying that the tertiary and primary structure of ALA was destroyed under simulated gastric digestion. The band that appeared near 14.4 kDa was completely undigested ALA. This observation confirmed that ALA was not completely digested because of the simulated immature gastric environment of infants [32]. Three bands were also discovered in ALA-0D, ALA-50D, and ALA-100D (Lanes 7–9 in Figure 1B), and the one near 3.3 kDa was smeared. This might account for further hydrolysis induced by trypsin and α-chymotrypsin accelerating the digestion of ALA-0D, ALA-50D, and ALA-100D, causing them to become loose and form small peptide fragments. Therefore, we suggested that gastric digestion destroyed the tertiary and primary structure of ALA-0, ALA-50, and ALA-100, and gastroduodenal digestion further damaged their tertiary and primary structure.

### 3.2. IgG/IgE-Binding Abilities and Western Blotting

**Figure 2:**
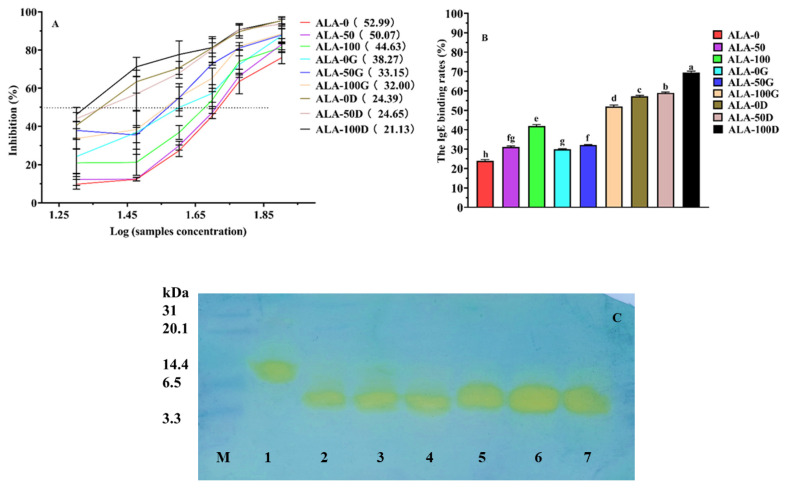
The IgG (**A**) and IgE (**B**) binding abilities of ALA treated by ultrasound during in-vitro gastric and gastroduodenal digestion determined by icELISA. Rabbit anti-ALA IgG was incubated separately with 20, 30, 40, 50, 60, 80 μg/mL substrate samples, gastric and gastroduodenal digesta. Letters (a–g) demonstrate significant differences (*p* < 0.05). Western blotting pattern (**C**) of ALA-0 (Lane 1), ALA-0G (Lane 2), ALA-50G (Lane 3), ALA-100G (Lane 4), ALA-0D (Lane 5), ALA-50D (Lane 6), and ALA-100D (Lane 7). The molecule weight distribution of marker (M) ranges from 3.3 to 31 kDa. IgG: immunoglobulin G, IgE: immunoglobulin E. ALA-0: untreated ALA, ALA-50: ultrasound-treated ALA at 50 W/cm^2^, ALA-100: ultrasound-treated ALA at 100 W/cm^2^. ALA-0G, ALA-50G, and ALA-100G: ALA-0, ALA-50, and ALA-100 digested by simulated gastric juice. ALA-0D, ALA-50D, and ALA-100D: ALA-0, ALA-50, and ALA-100 digested by simulated gastric juice followed simulated duodenal juice.

ALA is a major whey protein anaphylactogen that can bind with anaphylactic ALA-specific IgG/IgE, leading to various food anaphylactic symptoms. The IgG/IgE-binding abilities of substrate samples, gastric and gastroduodenal digesta were evaluated by icELISA and shown in Figure 2. Figure 2A shows the IgG IC_50_ value of ALA-50 and ALA-100 shifting to 50.07 and 44.63 μg/mL, respectively. However, the IgG IC_50_ value of ALA-0 was 52.99 μg/mL. Comparatively, 1.19 folds of the IgG IC_50_ value were observed when ALA was treated with the ultrasound intensity of 100 W/cm^2^. These results indicated that ultrasound unfolded the tertiary structure of ALA, and exposed the IgG-binding epitopes, resulting in increased anaphylaxis [23]. Furthermore, with the increase of ultrasound intensity, the tertiary structure of ALA gradually unfolded and exposed more IgG-binding epitopes, leading to higher anaphylaxis.

The IgG IC_50_ value of gastric digesta tended toward reduced values (increased anaphylaxis), compared with the substrate samples. The IgG IC_50_ value of ALA-0G was 38.27 μg/mL, and that of ALA-50G and ALA-100G successively declined when the ultrasound intensities were increased to 50 (33.15 μg/mL, 1.15 folds), and 100 W/cm^2^ (32.00 μg/mL, 1.20 folds), indicating an increase of IgG-binding ability in ALA-50G and ALA-100G. The tertiary structure of gastric digesta was destroyed after digestion (Figure 1B), so the conformational epitopes were destroyed. However, compared with the substrate samples, the anaphylaxis was enhanced in the gastric digesta, which probably explained that linear epitopes played a more important role in affecting the anaphylaxis of ALA, compared with the conformational epitopes. Furthermore, the presence of intact ALA molecules after digestion still remained strong anaphylaxis. Gazme et al. [33] showed that the in-vitro digestion caused the egg white proteins to remain residual immune-reactivity due to the presence of intact proteins such as lysozyme. The IgG IC_50_ value of ALA-0D (24.39 μg/mL), ALA-50D (24.65 μg/mL), and ALA-100D (21.13 μg/mL) was reduced again, and ALA-100D was reduced dramatically, with the highest anaphylaxis. 

Figure 2B showed the IgE-binding rates of the nine samples mentioned above, sharing similar trends such as the IgG-binding abilities. In general, the anaphylaxis of the substrate samples increased with the increase of ultrasound intensity, and the anaphylaxis of the gastric and gastroduodenal digesta was further increased, especially for gastroduodenal digesta. ALA-100, ALA-100G, and ALA-100D showed the highest IgE binding rates in substrate samples, gastric digesta, and gastroduodenal digesta (*p* < 0.05), respectively. Guimaraes et al. [34] found that Cry1Ab protoxin still conserved its immunoreactivity during digestion. 

Western blotting was executed to intuitively evaluate the IgG-binding abilities of ALA-0, ALA-0G, ALA-50G, ALA-100G, ALA-0D, ALA-50D, and ALA-100D, using self-made rabbit anti-ALA IgG (1:1000 dilution). HRP-labeled goat anti-rabbit IgG was diluted to 1:1000. Given the fact that the anaphylaxis of protein anaphylactogens increased after ultrasound treatment, only gastric and gastroduodenal digesta were embodied for western blotting analysis [30]. As shown in Figure 2C, in comparison with line 1 (ALA-0), lines 2–7 (ALA-0G, ALA-50G, ALA-100G, ALA-0D, ALA-50D, and ALA-100D) also bound with rabbit anti-ALA IgG, showing both digesta had strong anaphylaxis.

### 3.3. Bioactive Mediators Release Activity 

ALA would combine with fragment crystallizable (Fc) of the specific IgE that binds to the high-affinity IgE receptor FcεRI of mast cells or basophils during the effector phase, arousing cell degranulation and bioactive mediators, such as histamine, IL-6, and β-Hex generating, resulting in diarrhea, gastroduodenal bleeding, and other anaphylactic symptoms [35]. To investigate the influence of ultrasound on the release of bioactive mediators of ALA-0, ALA-50, ALA-100, and their digesta, KU812 cells were applied in the experiment. After the experimental KU812 cells were sensitized with CMA patients’ sera, FcεRI, a high-affinity IgE receptor that was believed to be closely related to cell degranulation, was expressed on the KU812 cells surface. The cells were then in contact with ALA-0, ALA-50, ALA-100, and their digesta, releasing bioactive mediators.

**Figure 3:**
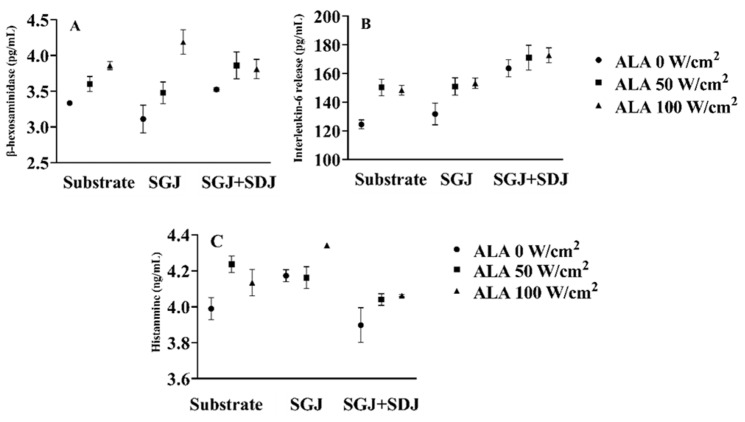
Influence of ultrasound treatment on the release of bioactive mediators. The release of β-Hex (**A**), the release of IL-6 (**B**), and the release of histamine (**C**). Substrate includes ALA-0, ALA-50, and ALA-100; SGJ is gastric digesta including ALA-0G, ALA-50G, and ALA-100G; SGJ+SDJ is gastroduodenal digesta containing ALA-0D, ALA-50D, and ALA-100D. SGJ: simulated gastric juice, SDJ: simulated duodenal juice. ALA-0: untreated ALA, ALA-50: ultrasound-treated ALA at 50 W/cm^2^, ALA-100: ultrasound-treated ALA at 100 W/cm^2^. ALA-0G, ALA-50G, and ALA-100G: ALA-0, ALA-50, and ALA-100 digested by simulated gastric juice. ALA-0D, ALA-50D, and ALA-100D: ALA-0, ALA-50, and ALA-100 digested by simulated gastric juice followed simulated duodenal juice.

Panels A, B, and C of Figure 3 show, respectively, the release of β-Hex, IL-6, and histamine of substrate samples, as well as those of their gastric and gastroduodenal digesta. Stimulating with ALA-0 caused KU812 cell degranulation, resulting in the release of β-Hex (3.33 pg/mL). Dramatically, ultrasound-treated ALA (ALA-50, ALA-100) showed increased degranulation ability and led to the increased release of β-Hex, demonstrating that the anaphylaxis was boosted via ultrasound treatment (Figure 3A). The results were consistent with the IgG/IgE-binding abilities analysis, which was due to the unfolded tertiary structure of ALA treated by ultrasound, leading to epitopes exposure. Besides, when simulated gastric digestion was carried out, the β-Hex release of gastric digesta were in the rank of ALA-0G (3.11 pg/mL) < ALA-50G (3.48 pg/mL) < ALA-100G (4.19 pg/mL), of which could also be authenticated in gastroduodenal digesta, increased release of β-Hex was discovered in the digesta of ultrasound-treated ALA. A similar phenomenon could be found in the release of IL-6 and histamine (Figure 3B,C). In general, the anaphylaxis of ultrasound-treated ALA was strengthened. We hypothesized the reason was that ultrasound unfolded the tertiary structure of ALA and exposed increased antibody-binding epitopes, thus promoting cell degranulation. The anaphylaxis of both the gastric/gastroduodenal digesta of ultrasound-treated ALA was still higher, compared with the gastric/gastroduodenal digesta of untreated ALA, further confirming that ultrasound increased the anaphylaxis of ALA during digestion.

### 3.4. Isolation and Anaphylaxis Analysis of Gastroduodenal Digesta Groups

**Figure 4:**
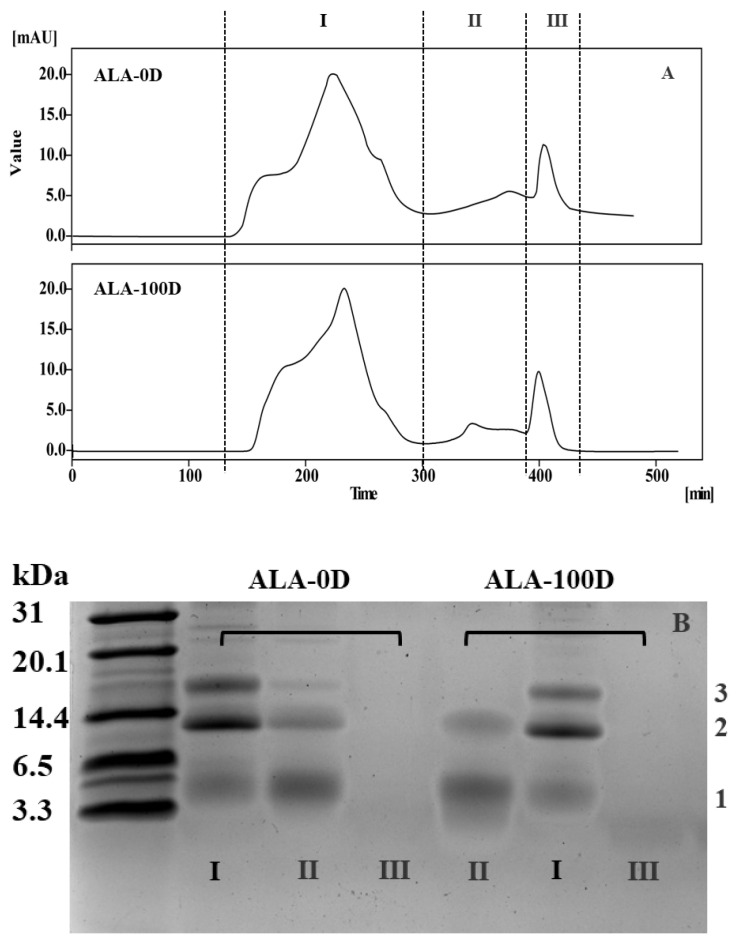
Isolation of ALA-0D and ALA-100D by Sephadex G-25 gel chromatography (**A**); the tricine SDS PAGE pattern of groups I, II, and III in ALA-100D and ALA-100D (**B**). ALA-0D and ALA-100D: untreated ALA and ultrasound-treated ALA at 100 W/cm^2^ digested by simulated gastric juice followed simulated duodenal juice.

We found that gastric/gastroduodenal digesta still had strong anaphylaxis, especially for ALA-100D. We therefore took ALA-100D as an example to isolate by gel filtration chromatography. ALA-0D and ALA-100D were isolated by the Sephadex G-25 column (Figure 4). Based on A_220_, the chromatogram was divided into three groups (I, II, and III), and the molecular weight of the three groups followed the order: group I > group II > group III. 

**Figure 5:**
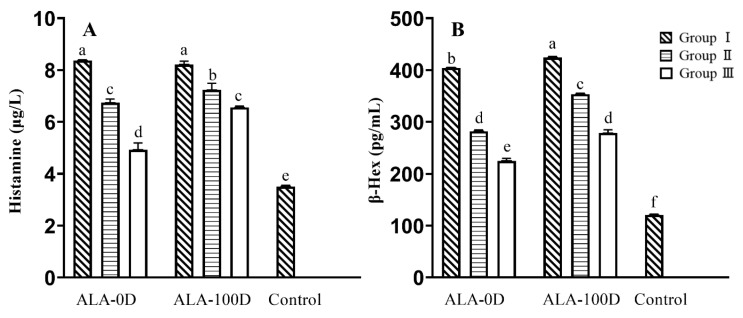
The KU812 cells degranulation of groups I, II, and III isolated from ALA-0D and ALA-100D. The release of histamine (**A**) and β-Hex (**B**). Letters (a–f) demonstrate significant differences (*p* < 0.05). ALA-0D and ALA-100D: untreated ALA and ultrasound-treated ALA at 100 W/cm^2^ digested by simulated gastric juice followed simulated duodenal juice.

The results in Figure 3 showed that the anaphylaxis increased after gastroduodenal digestion, and ultrasound treatment raised the anaphylaxis ability of ALA after gastroduodenal digestion. Therefore, the KU812 degranulation assay for the three groups (I, II, and III) in ALA-0D and ALA-100D was performed. The release of histamine and IL-6 of each group is shown in Figure 5. The histamine release of group I in ALA-0D and ALA-100D was 8.37 and 8.21 μg/L, and there was no significant difference between them. Compared with ALA-0D, the histamine release of group II in ALA-100D was 7.23 μg/L and higher (Figure 5A). A similar phenomenon was observed in group III in ALA-0D and ALA-100D. That was to say, the cell degranulation of group III in ALA-100D was higher than that in ALA-0D (*p* < 0.05). When the KU812 cells were exposed to group I in ALA-D, the β-Hex release was 404.15 pg/mL and was lower than that of ALA-100D (*p* < 0.05). Furthermore, when it came to group II and III, the β-Hex release content of ALA-100D was higher than ALA-0D (*p* < 0.05) (Figure 5B). In general, gastroduodenal digesta groups still retained anaphylaxis, and groups II and III had a low release activity of bioactive mediators. However, they still retained anaphylaxis.

### 3.5. Anti-Digestive Stable Peptides Identification

**Figure 6:**
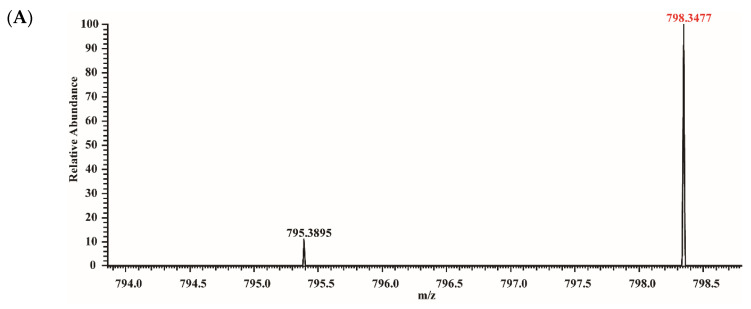

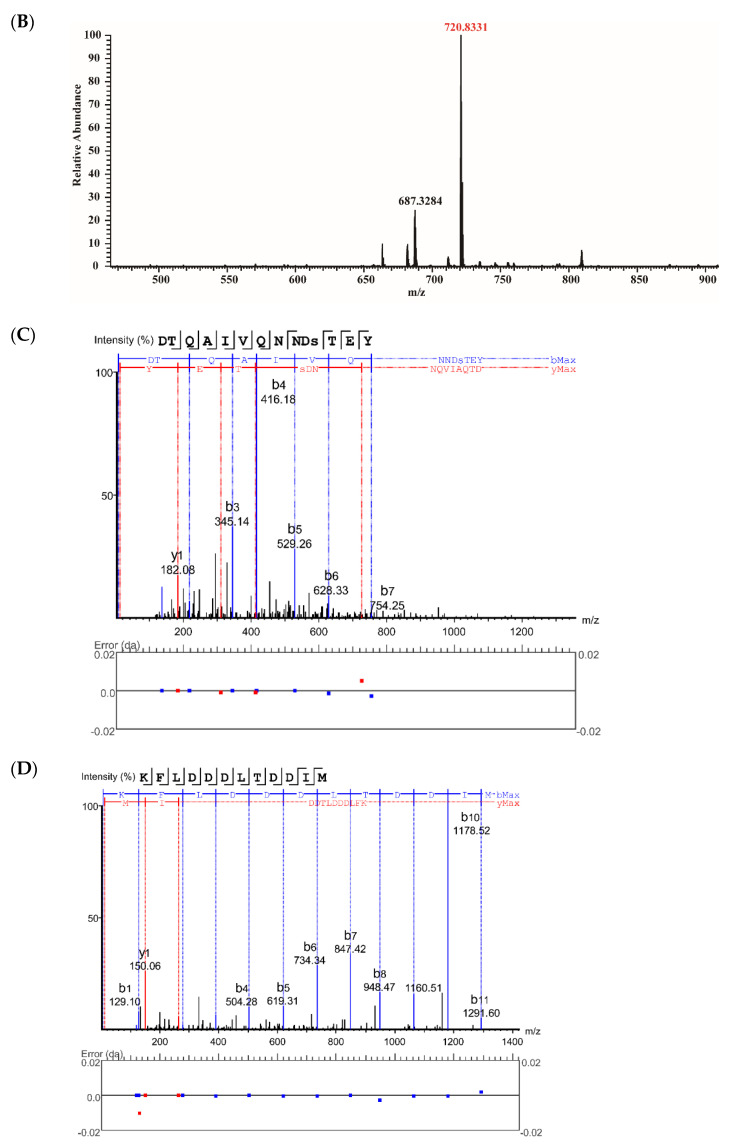
MS spectra of the anti-digestive stable peptides of DTQAIVQNNDSTEY and KFLDDDLTDDIM (**A**,**C**). HCD-MS/MS spectra of DTQAIVQNNDSTEY and KFLDDDLTDDIM (**B**,**D**).

**Figure 7:**
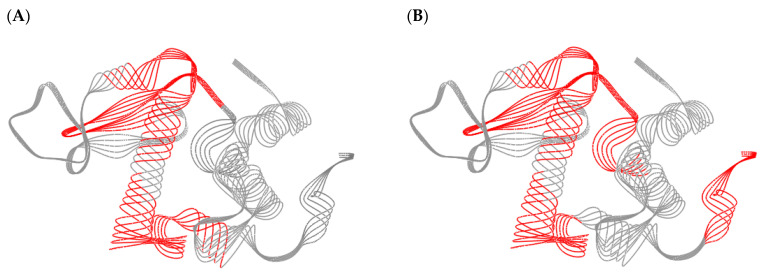
The 3D structure of ALA and the anti-digestive stable peptides of groups Ⅱ (**A**) and Ⅲ (**B**). The anti-digestive stable peptides are marked in red. Protein Data Bank (PDB) code: 1F6R. ALA: α-lactalbumin.

The anti-digestive stable peptides of groups II and III in ALA-0D and ALA-100D were analyzed by HPLC-MS/MS. Figure 6A-D shows the MS and MS/MS spectra of peptides DTQAIVQNNDSTEY (AA 37–50) and KFLDDDLTDDIM (AA 79–90). The *m*/*z* of AA 37–50 was 798.3477, and the peptide AA 37–50 was identified according to the difference between the values of b and y ions. The amino acid sequences of AA 79–90 and other peptides were identified in the same way. As shown in Table 1, it was found that 22 and 20 anti-digestive stable peptides were identified from group II in ALA-0D and ALA-100D, respectively, and identified peptides of group III in ALA-0D and ALA-100D were 16 and 10. However, it is difficult to assess if the differences on the number of identified peptides represent a significant difference on digestibility between ALA-0D and ALA-100D.These anti-digestive stable peptides of group II mainly distributed in 32–43 (32–43, 33–43, 36–43), 37–50 (37–44, 37–46, 37–49, 37–50, 37–49, 40–50, 41–50), 79–90 (79–90, 80–86, 80–90, 80–92, 81–90, 82–90, 84–90), 94–104 (94–103, 94–104, 95–103, 95–104, 96–103, 96–104), and 115–123. When it comes to group III, we found that the anti-digestive stable peptides mainly distributed in 37–50 (37–44, 37–46, 37–50, 38–50, 41–50), 79–90 (79–87, 79–90, 80–86, 80–90, 81–90, 82–90, 84–90), 94–104 (94–103, 94–104, 95–102, 95–103, 95–104, 96–104), 99–108 (99–107, 99–108). 99–107 (108) were only discovered in group Ⅲ of ALA-100D. This might be ultrasound unfolded the structure of ALA, leading peptide bond 98–99 and 107–108 (108–109) to be hydrolyzed and formed peptide 99–107 and 99–108. Figure 7 shows the anti-digestive stable peptides in the 3D structure of ALA. It could be seen that most of the anti-digestive stable peptides were distributed on the α-helix.

Jarvinen et al. [36] found linear epitopes of ALA including AA 1–16, AA 13–26, AA 47–58, AA 93–102, AA 7–18, AA 51–61, AA 89–108. The anti-digestive stable peptides such as AA 94–104 and AA 99–108 detected by us were located in AA 93–102 and AA 89–108. Maynard et al. [37] identified AA 99–108, AA 17–58, AA 109–123, and AA 59–94 as the linear epitopes of ALA, and AA 37–50, AA 79–90 and AA 115–123 detected by us overlapped with some of the epitopes they identified. Li. et al. [38] found that linear epitopes of ALA were AA 37–46, AA 52–54, AA 56–59, and AA 63–72, AA 74–76, AA 81–90, AA 92–99, AA 41–46, AA 55–60, AA 62–72, AA 74–76, AA 85–90 and AA 92–99, and the linear epitopes such as AA 41–46, AA 85–90, AA 37–46, AA 81–90 remained intact on the anti-digestive stable peptides (AA 41–50, AA 80–92, AA 80–95, AA 81–90, AA 37–50, AA 81–90). Some linear epitopes are still retained on the anti-digestive stable peptides produced after gastroduodenal digestion, and these linear epitopes are exposed, resulting in increased anaphylaxis after digestion.

## 4. Conclusions

In this work, it was exhibited that ultrasound unfolded the structure of ALA, thereby increasing the IgG/IgE-binding abilities and also raising the release of the bioactive mediators. The anaphylaxis of untreated and ultrasound-treated ALA further increased after digestion, which might be that linear epitopes play an important role in affecting anaphylaxis compared with the conformational epitopes. Furthermore, some linear epitopes were still retained on the anti-digestive stable peptides produced after gastroduodenal digestion. Among gastric/gastroduodenal digesta, the digesta of ultrasound-treated ALA embodied high anaphylaxis. Therefore, ultrasound could increase the anaphylaxis of ALA during digestion.

## Figures and Tables

**Table 1:** The anti-digestive stable peptides determined by high-performance liquid chromatography system coupled to tandem mass spectrometry (LC-MS/MS).

Peptide	Mass	Length	ppm	*m*/*z*	Start	End
**Group II in ALA-0D**						
GGVSLPEWV	942.4811	9	1.4	472.2485	19	27
TSGYDTQAIVQ	1181.5564	11	1.6	591.7864	33	43
YDTQAIVQ	936.4553	8	0.2	469.235	36	43
DTQAIVQN	909.4168	8	0.1	455.7157	37	44
DTQAIVQNND	1099.4781	10	1.5	550.7472	37	46
DTQAIVQNNDSTE	1433.6271	13	2.4	717.8225	37	49
DTQAIVQNNDSTEY	1618.6722	14	0.1	810.3435	37	50
AIVQNNDSTEY	1274.5391	11	−0.2	638.2767	40	50
IVQNNDSTEY	1181.52	10	−0.4	1182.5269	41	50
KFLDDDLTDDIM	1421.6384	12	2.5	711.8282	79	90
FLDDDLT	859.3575	7	0.3	430.6862	80	86
FLDDDLTDDI	1162.5029	10	0.3	582.2589	80	89
FLDDDLTDDIM	1333.536	11	0.1	667.7753	80	90
FLDDDLTDDIMCV	1513.6316	13	0.1	757.8231	80	92
FLDDDLTDDIMCVKKI	1966.9268	16	0.3	984.4709	80	95
LDDDLTDDIM	1146.4751	10	0.8	574.2453	81	90
DDDLTDDIM	1073.3835	9	1.9	537.7001	82	90
KILDKVGINY	1161.6758	10	−0.1	581.8451	94	103
ILDKVGINY	1055.5627	9	0.7	1056.5708	95	103
ILDKVGINYW	1219.66	10	1	610.8379	95	104
LDKVGINY	920.4967	8	1	461.2561	96	103
LDQWLCEKL	1146.5743	9	1.8	574.2955	115	123
**Group III in ALA-0D**						
HTSGYDTQ	907.3672	8	1.7	454.6906	32	39
DTQAIVQN	869.4243	8	3	435.7197	37	44
DTQAIVQNND	1138.4867	10	−0.4	570.249	37	46
DTQAIVQNNDSTEY	1618.6722	14	−0.1	810.3413	37	50
TQAIVQNNDSTEY	1498.6899	13	3	750.3527	38	50
IVQNNDSTEY	1203.502	10	0.5	602.7571	41	50
GLFQINNK	932.5079	8	3.1	467.2615	51	58
KFLDDDLTD	1080.4974	9	4.9	541.2573	79	87
KFLDDDLTDDIM	1455.6439	12	0.3	728.8277	79	90
FLDDDLTDDIM	1333.536	11	0	667.7736	80	90
FLDDDLTDDIMCV	1513.6316	13	2.3	757.823	80	92
LDDDLTDDIM	1180.4806	10	3.2	591.248	81	90
DDDLTDDIM	1051.4016	9	3.2	526.7085	82	90
DLTDDIM	821.3477	7	3.6	822.3559	84	90
LDKVGIN	870.5175	8	1.7	436.2657	95	102
ILDKVGINYW	1220.644	10	4.7	611.3307	95	104
**Group II in ALA-100D**						
ELKDLKGY	946.5123	8	1.1	474.2639	11	18
HTSGYDTQAIVQ	1319.5994	12	−0.3	660.8068	32	43
TSGYDTQAIVQ	1181.5564	11	1.5	591.7864	33	43
DTQAIVQN	888.4189	8	0.9	445.2171	37	44
DTQAIVQNND	1117.4888	10	0.4	559.7519	37	46
DTQAIVQNNDSTE	1415.6165	13	−0.9	708.8149	37	49
DTQAIVQNNDSTEY	1634.6373	14	0	818.3259	37	50
TQAIVQNNDSTEY	1481.6635	13	1.4	1482.6727	38	50
AIVQNNDSTEY	1274.5391	11	0.1	638.2769	40	50
IVQNNDSTEY	1203.502	10	−0.4	602.758	41	50
KFLDDDLTDDIM	1455.6439	12	1.5	728.8303	79	90
FLDDDLTDDIM	1333.536	11	0.1	667.7753	80	90
FLDDDLTDDIMCV	1529.6266	13	−2.2	765.8189	80	92
LDDDLTDDIM	1180.4806	10	0.7	591.248	81	90
KILDKVGINY	1183.6577	10	−0.4	1184.6646	94	103
KILDKVGINYW	1369.7369	11	0.6	685.8762	94	104
ILDKVGINYW	1219.66	10	1.1	610.838	95	104
ILDKVGINY	1033.5808	9	0.7	517.798	95	103
LDKVGINY	921.4807	8	0.2	461.7477	96	103
LDQWLCEKL	1146.5743	9	0.4	574.2947	115	123
**Group III in ALA-100D**						
GYGGVSLPEWV	1162.5658	11	1.3	582.291	17	27
DTQAIVQNNDSTEY	1618.6722	14	1.8	810.3448	37	50
LDDDLTDDIM	1333.536	11	0	667.7753	80	90
KILDKVGINY	1143.6652	10	1.4	572.8406	94	103
KILDKVGINYW	1363.75	11	−0.6	682.8818	94	104
ILDKVGINY	1032.5968	9	1.2	517.3063	95	103
ILDKVGINYW	1235.655	10	0.7	618.8352	95	104
LDKVGINYW	1106.576	9	0	554.2953	96	104
VGINYWLAH	1071.5502	9	0.7	536.7827	99	107
VGINYWLAHK	1200.6292	10	0.8	601.3223	99	108

## Supplementary Materials

The following are available online at https://www.mdpi.com/article/10.3390/foods10112760/s1, Table S1: The information of CMA patients’ sera.
