# Supplementary material for: Investigation on the Anaphylaxis and Anti-Digestive Stable Peptides Identification of Ultrasound-Treated α-Lactalbumin during In-Vitro Gastroduodenal Digestion
Source: Foods. 2021 Nov 10;10(11):2760. doi: 10.3390/foods10112760 (PMC8623493; doi:10.3390/foods10112760)
Supplement: Supplementary file 1 [file foods-10-02760-s001.zip › foods-1448484-supplementary.pdf]

Table S1 The information of CMA patients' sera

Patients	Gender	Ages	Bleed number	IgE activity (kU/L)	Clinical history
1	Male	27	PL 26529	10.774	Allergic rhinitis
2	Male	18	PL 26449	5.38	Allergic rhinitis, multi-allergic
3	Female	27	PL 26248	20.818	Allergic rhinitis
4	Female	40	PL 26046	14.6	Allergic rhinitis
5	Male	26	PL 23861	9.12	Allergic rhinitis, multi-allergic
6	Male	29	PL 23320	91	Allergic rhinitis
7	Male	33	PL 26666	45.4	Allergic rhinitis
8	Other	24	PL 24748	≥100	Allergic rhinitis
9	Female	66	PL 27061	70.5	Allergic rhinitis, multi-allergic
10	Male	22	PL 20838	30.5	Allergic rhinitis
